# HRQoL improves in treatment-naïve HIV-1 subjects initiated on lopinavir/ritonavir (LPV/r) with raltegravir (RAL) or tenofovir/emtricitabine (TDF/FTC)

**DOI:** 10.1186/1758-2652-13-S4-P8

**Published:** 2010-11-08

**Authors:** RW Baran, B Dietz, LM Fredrick, M Tian, T Podsadecki

**Affiliations:** 1 Abbott Laboratories, Global Health Economics & Outcomes Research, Abbott Park, USA; 2 Abbott GmbH & Co. KG, Global Health Economics & Outcomes Research, Ludwigshafen, Germany; 3 Abbott Laboratories, Global Statistics, Abbott Park, USA; 4 Abbott Laboratories, Global Pharmaceutical Research and Development, Abbott Park, USA

## Purpose

The clinical status of HIV-1 infected patients initiated on modern anti-retroviral (ARV) therapy is consistently improved as indicated by reduced viral load (VL) and increased CD4+ T-cell count, however, the reported impact on patients' health related quality of life (HRQoL) has been variable. An appropriate assessment of HRQoL response over time in ARV-naïve HIV-1 infected subjects initiated on LPV/r combined with RAL or TDF/FTC could reveal the impact of these therapies on functional status and wellbeing.

## Methods

The PROGRESS study is an ongoing, randomized, open-label 96-week trial of LPV/r 400/100 mg BID combined with either RAL 400 mg BID (n=101) or TDF/FTC 300/200 mg QD (n=105) in ARV-naïve subjects. Subjects completed the MOS-HIV, a validated, disease-specific HRQoL instrument, at baseline and weeks 8, 24, and 48. The MOS-HIV comprises 35 items in eleven dimensions. Dimension specific scores and the two summary scores (Physical Component Summary [PCS], Mental Component Summary [MCS]) each range from 0 to 100 points, with higher scores indicating better function or well being. Changes in score from baseline were analyzed using ANCOVA with the following covariates: baseline score, treatment arm, gender, race/ethnicity, age, time since HIV-1 diagnosis, baseline CD4+ T-cell count, and plasma HIV-1 RNA level (VL).

## Results

Both LPV/r + RAL and LPV/r + TDF/FTC treatment arms achieved similar VL and CD4+ T-cell endpoints at 48 weeks; a similar proportion of subjects in each arm discontinued the study prematurely. For each assessment period, >79% of all subjects completed the MOS-HIV survey. There were no statistically significant differences between treatment arms in MOS-HIV scores on any dimension or summary score at any assessment period (p>0.100). When pooling data across all subjects, MCS improved significantly from baseline at week 8 (mean change: +2.3, p = 0.013), week 24 (+3.5, p<0.001), and week 48 (+2.2, p=0.041). PCS improved significantly from baseline only at week 24 (+1.8, p=0.033). The General Health Perceptions dimension, an overall evaluation of health, was significantly improved at week 8 (+5.4, p= 0.009), week 24 (+7.9, p<0.001), and week 48 (+6.0, p=0.019).

## Conclusions

LPV/r in combination with either RAL or TDF/FTC improved HRQoL related to both mental and physical states as well as overall health in ARV naïve, HIV-1 infected subjects at weeks 8, 24, and 48. Additional HRQoL data through week 96 are being collected.

